# An increased fraction of circulating miR-363 and miR-16 is particle bound in patients with chronic lymphocytic leukaemia as compared to normal subjects

**DOI:** 10.1186/s13104-018-3391-9

**Published:** 2018-05-08

**Authors:** Afaf Alharthi, Daniel Beck, Dena R. Howard, Peter Hillmen, Melanie Oates, Andrew Pettitt, Simon D. Wagner

**Affiliations:** 1 Leicester Cancer Research Centre and Ernest and Helen Scott Haematological Research Unit, University of Leicester, Room 104, Hodgkin Building, Lancaster Road, Leicester, LE1 7HB UK; 2 St James Institute of Oncology, Leeds, UK; 3 University of Liverpool, Level 6, Duncan Building, Daulby Street, Liverpool, L69 3GA UK

**Keywords:** Chronic lymphocytic leukaemia, miRNA, Extracellular vesicle

## Abstract

**Objectives:**

In vitro culture studies have shown that miR-363 is enriched in extracellular vesicles from chronic lymphocytic leukaemia cells. We wondered whether miR-363 was detectable in plasma, which is an essential precondition for further studies to assess its usefulness as a biomarker. Using samples from two clinical trials: one enrolling patients with advanced disease and the other asymptomatic patients with early stage disease, we determined plasma miR-363 levels and secondly investigated the distribution of this miRNA between plasma and particle bound fractions in patients and normal subjects.

**Results:**

Advanced disease (n = 95) was associated with higher levels of miR-363 than early stage disease (n = 45) or normal subjects (n = 11) but there was no association with markers of prognosis. The distribution of specific miRNA between particle bound and plasma protein fractions was investigated using size exclusion chromatography on plasma from patients (n = 4) and normal subjects (n = 3). ~ 20% of total miR-16 and miR-363 is particle bound in patients while there was no detectable particle bound material in normal subjects. Our work demonstrates that miR-363 levels are raised in chronic lymphocytic leukaemia patients and raises the possibility that distribution of circulating miRNA between plasma fractions differs in health and disease.

## Introduction

MiRNA in plasma associate with either particles or plasma protein fractions [1]. In plasma extracellular vesicles (EV), defined as particles ≤ 1 µm in diameter, are heterogeneous and comprise exosomes, which have a diameter ~ 100 nm, and are derived through fusion of multi-vesicular bodies with the plasma membrane and the larger microvesicles, which might represent blebs derived from the plasma membrane [2]. Exosomes carry a cargo of protein, mRNA and miRNA and have established roles in normal innate and acquired immunity and in cancer biology [3–6] and exert some of their effects through transfer of their cargo (protein, mRNA and miRNA) from one cell to another [7, 8].

Intracellular levels of specific miRNA predict clinical outcome in the low grade B cell lymphoproliferative disorder, chronic lymphocytic leukaemia (CLL) [9]. MiRNA signatures that allow distinction between CLL patients and normal subjects have also been determined [10, 11]. Circulating miRNA have been detected in CLL patients and one study suggested that miR-363 levels were elevated in this disease but not other lymphoproliferative conditions i.e. hairy cell leukaemia or myeloma [10]. Measurement of circulating miR-363 from a small number of patients suggested that levels of this miRNA increased in line with increasing clinical stage [10]. Others have focused on characterising EV miRNA and demonstrated a CLL-specific signature [11].

Proliferation of B-cells is driven by two major pathways, firstly through the B-cell receptor following engagement by antigen [11] and secondly through CD40, following activation by CD40L, a T-cell surface marker [12]. Both routes enhance EV secretion by CLL cells. EVs produced by CD40L stimulated CLL cells are enriched in miR-363, suggesting that this miRNA is specifically selected into the particles [12]. EVs from CD40L stimulated CLL cells, when taken up by autologous CD4^+^ helper T-cells in vitro, perturb immune synapse formation and T-cell motility [12] and miR-363 knockdown in CLL cells altered the effects of CLL EVs on T-cells. This work suggests that EVs produced by CLL cells in the tumour microenvironment (TME) have roles in communication with either T-cells or, as suggested by others, stromal cells [13, 14]. The TME is the site at which CLL cells proliferate and survive to resist the effects of chemotherapy [15]. A hypothesis generated by these studies is that miRNA enriched in EVs produced in the TME might be biomarkers of disease activity or response to therapy if detectable in the circulation.

Because miRNA are detectable in the circulation it is believed that they have potential as readily measurable biomarkers in cancer [16–18] and indeed there may be disease specific signatures for each type of tumour [17]. In order to investigate the potential of miR-363 as a biomarker from the TME we firstly determined plasma levels and association with prognostic markers and secondly, investigated the distribution between particle and plasma protein fractions.

## Main text

### Materials and methods

#### Patient samples

Plasma samples from normal subjects (n = 11) were obtained after informed consent was obtained (Leicester Research Ethics Committee 06/Q2501/122). Plasma samples were obtained through the UK CLL Trials Biobank (University of Liverpool) (North-West England Research Ethics Committee 14/NW/1014) from CLL patients enrolled in two clinical trials: the ARCTIC trial which was funded by the NIHR Health Technology Assessment Programme (NIHR HTA project number 07/01/38; ISRCTN16544962) (University of Leeds) [19] (n = 100) and CLEAR [A trial looking at using antibiotics for chronic lymphocytic leukaemia (http://www.cancerresearchuk.org/about-cancer/find-a-clinical-trial/a-trial-looking-using-antibiotics-for-chronic-lymphocytic-leukaemia-the-clear-trial)] (n = 50). For ARCTIC, a trial investigating advanced disease requiring treatment, median age was 63 years, interquartile range 58–67 years and M:F was 69:31. 48 patients had unmutated immunoglobulin genes, 36 mutated and 16 not determined. 14 patients showed 11q23 deletion and 4 patients 17p deletion by FISH interphase cytogenetics. Clinical information has not yet become available for patients enrolled in CLEAR, a trial enrolling asymptomatic patients with early stage disease. It was not possible to complete processing of 5 ARCTIC samples and 2 CLEAR samples, either because miRNA isolation failed or RT-PCR failed, and these cases were, therefore, excluded from the study.

#### Size exclusion chromatography

An ÄKTA Prime (GE Healthcare, Little Chalfont, UK) with a sephacryl S-500 resin chromatography column (0.9 × 30 cm, 19.1 ml bed volume) was employed to fractionate plasma samples. Before injection, the column was equilibrated with phosphate buffered saline (PBS) (pH 7.4) (25 ml) solution at 0.5 ml/min at room temperature. Platelets were depleted from fresh plasma by two rounds of centrifugation. The sephacryl column was then injected with 7 ml of undiluted plasma and eluted at room temperature for approximately 1 h with PBS solution at a flow rate of 0.5 ml/min. A total of 31–37 fractions of 4 ml each were collected. The column was flushed with 75 ml of PBS solution at 0.5 ml/min (3.75 column volumes) between plasma fractionation to eliminate carryover. Protein molecular weight standard BSA (67 kDa; GE Healthcare) was used. Fractions were stored at 4 °C before use.

#### MiRNA isolation and quantitative RT-PCR

QIAzol Lysis Reagent (Qiagen, Hilden, Germany, Cat No. 79306) was added to ultra-centrifuged plasma samples. The upper, aqueous phase was extracted, and ethanol was added to provide appropriate binding conditions for all RNA molecules from approximately 18 nucleotides (nt) upwards. The sample was then applied to the RNeasy MinElute spin column (MiRNeasy Kit, Qiagen, Cat No. 217004) and RNA eluted in RNase-free water.

To assess recovery and stability of RNA, each sample was spiked with an identical amount of synthetic UniSp2 RNA (Exiqon, Vedbaek, Denmark, #203203). Patient samples were taken into heparinised tubes but heparin is an inhibitor of enzymatic reactions. Therefore, plasma samples were treated with heparinase I (H2519; Sigma, St. Louis, MO, USA) according to the manufacturer’s instructions. Extracted miRNA were then reverse transcribed using Universal cDNA synthesis kit (Exiqon, #203301) according to the manufacturer’s protocol. The template RNA samples were diluted to a concentration of 40 ng/µl using nuclease free water. The reaction was incubated for 60 min at 42 °C before termination.

**Fig. 1:**
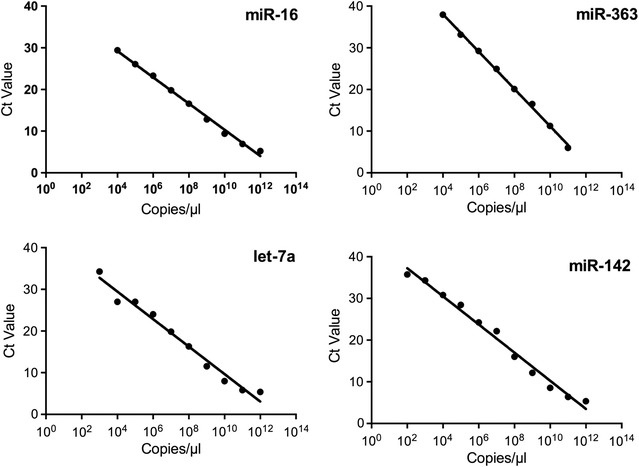
Standard curves for quantitative RT-PCR. Standard curves were constructed for mir-363, miR-16, miR142 and let-7a. Using known numbers of copies of each oligonucleotide as template RT-PCR was carried out and Ct values obtained. Trend line was interpolated using GraphPad Prism v6.0

Standard curves (Fig. 1) were constructed using synthetic oligonucleotide templates (Sigma, St. Louis, MO, USA) for miR-16 (UAGCAGCACGUAAAUAUUGGCG), miR-363-3p (5′-AAUUGCACGGUAUCCAUCUGUA), miR-142-3p (5′-UGUAGUGUUUCCUACUUUAUGGA) and let-7a-5p (5′-UGAGGUAGUAGGUUGUAUAGUU) (ThermoFisher, Waltham, MA, USA).

Quantitative PCR reactions were performed using SYBR green and miRNA-specific primers (Exiqon, hsa-miR-363-3p LNA PCR primer set #204726, hsa-miR-142-3p LNA PCR primer set #204291, hsa-let-7a-5p LNA PCR primer set #206084, hsa-miR-16-5p LNA PCR primer set #205702) according to the manufacturer’s instructions. cDNA produced in the RT reaction was amplified in MicroAmpTM optical 96-well reaction plates in triplicate 10 µl reactions on an Applied Biosystems 7900HT Thermocycler. Concentration and quality of nucleic acids were checked using NanoDrop^®^ ND-1000 spectrophotometer (NanoDrop Technologies, Wilmington DE, USA).

### Results

#### Plasma MiR-363 levels are elevated in patients as compared to normal subjects

**Fig. 2:**
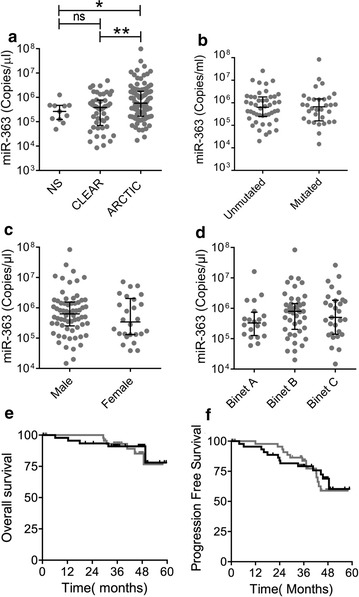
MiR-363 levels and clinical outcome. **a** Mir-363 levels are compared between healthy volunteers (HV) (n = 11), asymptomatic patients enrolled in the CLEAR clinical trial (n = 48) and patients, who met the criteria for treament, enrolled in the ARCTIC clinical trial (n = 95). Mean ± SEM are indicated. There was no significant difference (Mann–Whitney test) between HVs and CLEAR patients but there were significant differences between HVs and ARCTIC (*P *= 0.0313) patients and between CLEAR and ARCTIC patients (*P *= 0.0091). **b** MiR-363 levels of ARCTIC patients with mutated and unmutated immunoglobulin heavy chain genes are compared. Mean ± SEM are indicated. There was no significant difference (Mann–Whitney test) between groups. **c** MiR-363 levels of ARCTIC patients are compared by gender. Median and interquartile ranges are indicated. There was no significant difference (Mann–Whitney test) between groups. **d** MiR-363 levels of ARCTIC patients are compared by Binet clinical stage. Binet A indicates progressive stage A disease. Median and interquartile ranges are indicated. There was no significant difference (Mann–Whitney test) between groups. **e**, **f** Kaplan–Meier survival curves of ARCTIC patients. Patients were grouped into those with miR-363 levels above the median (black line) and those with levels below the median (grey line). There was no significant difference (Log-Rank (Mantel-Cox) test) in **e** overall survival or **f** progression free survival

Levels of miR-363 were compared between normal subjects (n = 11) and CLL patients with early stage (CLEAR) (n = 48) or advanced (ARCTIC) disease (n = 95). Levels in patients vary over ~ 1000-fold, from 10^4^ to 10^7^ copies/µl, and were significantly higher in patients with advanced disease as compared to patients with early stage disease (*P *= 0.0091, Mann–Whitney test) or normal subjects (*P *= 0.0313) (Fig. 2a) while there was no significant difference between normal subjects and patients with early stage disease. Immunoglobulin gene mutational status is an established CLL prognostic marker but for patients with advanced disease there was no significant difference in miR-363 levels between those with mutated or unmutated immunoglobulin genes (Fig. 2b). Cytogenetic aberrations are associated with clinical outcome. Deletion of 17p and 11q23 are both associated with reduced overall survival as compared to patients with normal karyotypes [20]. Analysis of ARCTIC data showed there was no significant difference in miR-363 levels in patients with either 17p deletion (n = 4) or 11q23 (n = 14) deletion as compared to those with a normal karyotype. Similarly neither gender nor Binet clinical stage were associated with significant differences in amounts of miR-363 (Fig. 2c, d).

We compared outcomes for patients with miR-363 levels greater than the median for the group with those whose miR-363 level was less than that of the median (Fig. 2e, f). There was no significant difference between these groups in either overall or progression free survival.

Therefore, miR-363 levels are higher in patients with advanced disease but there is no association between higher levels and prognostic markers or clinical outcome.

#### Circulating miR-363 distribution between plasma protein and particle bound fractions

**Fig. 3:**
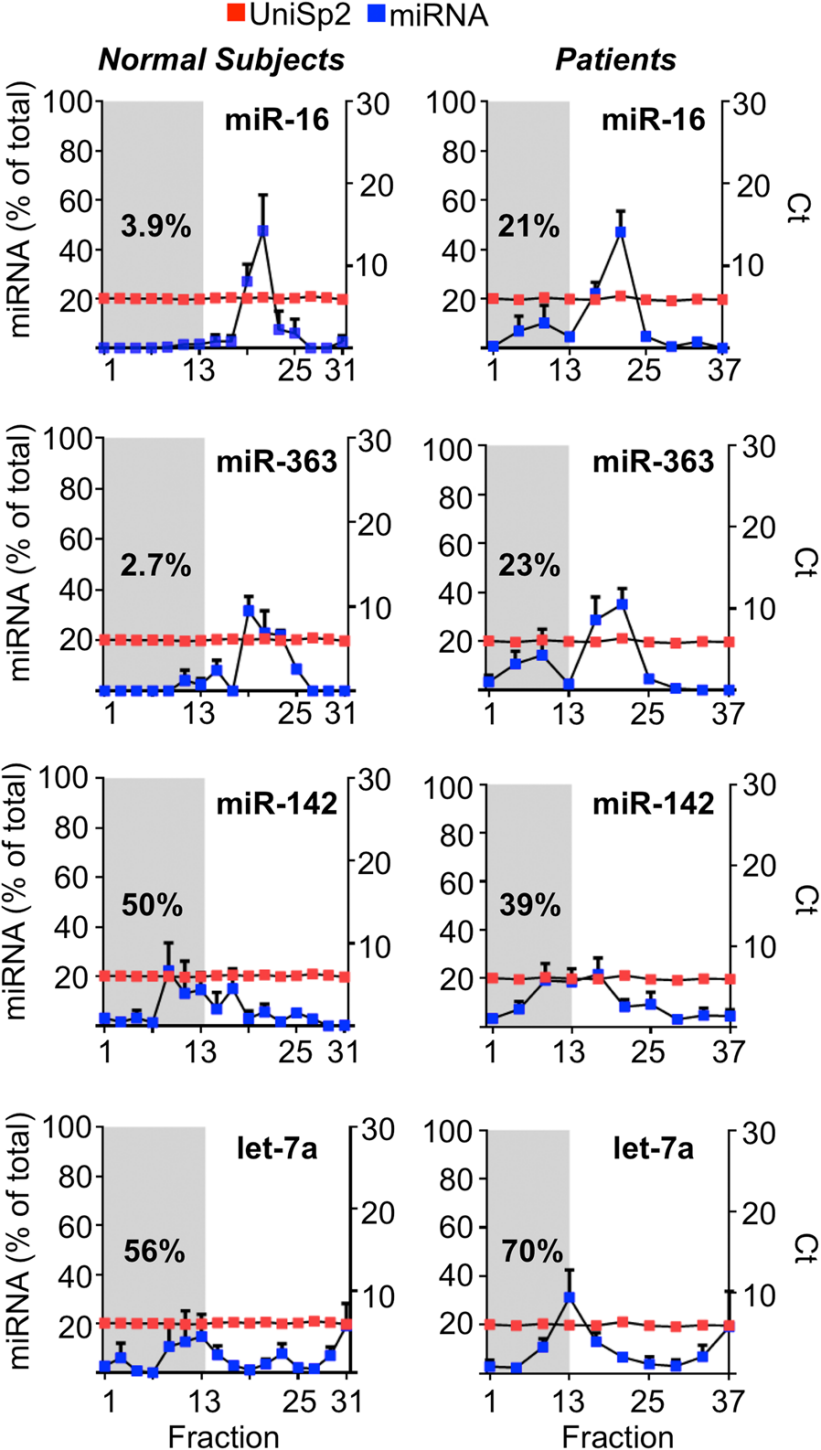
Distribution of selected miRNA between plasma protein and particle bound fractions of plasma. Between 31 and 37 plasma fractions were obtained by size exclusion chromatography from either healthy volunteers (panels to the left) or patients (panels to the right) as indicated on the x-axis. Fractions 1–13 are designated early particle containing fractions (grey shaded area). UniSp2 RNA spike-in was employed as a control and quantitative RT-PCR Ct values obtained are shown (red squares and right-y-axis). For each individual miRNA tested (mir-363, miR-16, miR142 and let-7a) the percentage of the total amount of miRNA in each fraction is plotted (blue squares and left-y-axis) of each individual graph. Percentage of total within fractions 1–13 is presented within the grey shaded area

Others have shown that, in single donors, miR-16 and miR-363 co-fractionate with plasma protein fractions whereas let-7a and miR-142 co-fractionate with large protein complex/particle fractions [1]. We wished to investigate miR-363 because of our previous work including having established that there are higher circulating levels in patients as compared to normal subjects. Based on the work of Arroyo et al. [1] miR-16 acted as a control for plasma protein bound miRNA whereas miR-142 and let-7a were controls for more particle bound miRNA. Total amounts of let-7a and miR-142 (0.8 × 10^4^ ± 0.3 × 10^4^ and 0.3 × 10^4^ ± 0.08 × 10^4^ copies/µl respectively, mean ± SEM) were much lower than for miR-16 (4.1 × 10^4^ ± 2.6 × 10^4^ copies/µl). In normal subjects miR-363 was highly expressed (10.6 × 10^4^ ± 4.6 × 10^4^ copies/µl) and was detectable in the small protein fractions (97% in fractions 13–31) (Fig. 3). As expected there were greater amounts of total miRNA in patients: miR-16 (30.5 × 10^4^ ± 14.8 × 10^4^ copies/µl) and miR-363 (55.0 × 10^4^ ± 23.7 × 10^4^ copies/µl) and to a lesser extent for miR-142 (1.8 × 10^4^ ± 0.8 × 10^4^ copies/µl) and let7a (1.3 × 10^4^ ± 0.4 × 10^4^ copies/µl).

Increased levels of miR-363 due to EV release by activated CLL cells in the TME might lead to an increased proportion of circulating and particle bound miRNA. In order to determine the distribution of miR-363 in patients (n = 4) and normal subjects (n = 3) between particle bound and plasma protein fractions we carried out size exclusion chromatography followed by quantitative RT-PCR.

We confirmed that in normal subjects miR-16 co-fractionated with the plasma protein fractions (96% in fractions 14–31) (Fig. 3) but let-7a and miR-142 were distributed more evenly across particle bound and plasma protein fractions (50 and 56% respectively in fractions 1–13). There were significant differences in distribution in patients as compared to normal subjects. Patients showed relatively more miRNA in the early eluting large protein/particle fractions than normal subjects. For miR-16 21% was in fractions 1–13, which is significantly more than in the later fractions (*P *= 0.0061, Mann–Whitney test) and similarly for miR-363 23% was present in the early fractions, which was again significantly more than in the later fractions (*P *= 0.033). These differences were not observed for miR-142 or let-7a.

### Discussion

There is a wealth of data to show that the tumour microenvironment (TME), which for CLL can be either lymph node or bone marrow, is essential for driving leukemic cell proliferation and mediates survival in the face of chemotherapy. By contrast circulating leukemic cells are predominantly non-dividing and quiescent. A reasonable hypothesis is that a marker of leukemic cell activity in the TME will be useful in guiding clinical decisions.

Our focus has been on miRNA, oligonucleotides with essential roles in regulating gene expression [21]. MiRNA are part of the cargo of EVs and we [12] and others [22] have proposed that they mediate some aspects of intercellular communication in the TME. MiRNA are also readily detectable in the blood and patterns of miRNA can be diagnostic for specific cancers [23] including CLL [11]. Circulating miRNA also hold promise as predictive biomarkers in cancer [24, 25] and in CLL [10].

Others have investigated the distribution of specific miRNAs between plasma protein and particle bound fractions [1]. In the normal subjects that these authors investigated the majority of miRNA were found in the plasma protein and not the particle fractions and they demonstrate that Argonaute2 co-purifies and stabilises these miRNA. A minority of miRNA, in normal subjects, associated with particles.

We focused on miR-363 because our work suggested enrichment in EVs following stimulation of leukemic cells by CD40L/IL-4 [26] and that particle bound miR-363 perturbs several functions of autologous CD4^+^ T-cells in vitro [12]. Others showed, in a small number of patients, that circulating miR-363 levels associated with clinical stage of CLL [10]. In normal subjects miR-363 appears to belong to the majority of miRNA that are predominantly in plasma protein fractions.

Our study is the first to investigate changes in the distribution of miRNA between protein and particle fractions of plasma in a disease. Four miRNA were investigated miR-363, miR-142, miR-16 and let-7a. In addition to miR-363, miR-16 is elevated in the plasma of CLL patients as compared to normal subjects (or patients with myeloma or hairy cell leukemia) [10] and might have a function in the development of this condition [27]. Like miR-363 in normal subjects miR-16 is predominantly in plasma protein fractions [1] but let-7a and miR-142 are mostly present in the particle fractions. In our group of normal subjects we demonstrate presence of the majority of miR-363 and miR-16 in plasma protein fractions confirming the previous work [1]. We did not find a clear separation of miR-142 or let-7a between plasma protein and particle fractions in normal subjects, although at least 50% was present in particle bound fractions. However, patients show a clear increase in levels of particle bound miR-363 and miR-16, which is not observed for miR-142 or let-7a.

We speculate that enrichment of miR-363 and miR-16 in EVs from CLL cells in the TME is reflected in increased circulating and particle bound miRNA in patients as compared to normal subjects. This represents a new parameter for defining differences between normal subjects and patients and it will be interesting to discover if this principle applies to other cancers.

## Limitations

We found a > 1000-fold difference in plasma levels of miRNA. Biological heterogeneity is likely to contribute to this but unavoidable differences in sample processing might also play a part. We made every effort to process samples from normal subjects and patients in exactly the same manner but it is known that sample handling can affect the results of miRNA assays [28] and there is unavoidable variability in the collection of patient samples.

## Abbreviations

CLL: chronic lymphocytic leukaemia
EV: extracellular vesicles
FISH: fluorescence in situ hybridisation
PBS: phosphate buffered saline
TME: tumour microenvironment
